# Venom from *Loxosceles* Spiders Collected in Southeastern and Northeastern Brazilian Regions Cause Hemotoxic Effects on Human Blood Components

**DOI:** 10.3390/toxins16120532

**Published:** 2024-12-10

**Authors:** Rafaela Silva-Magalhães, Ayla Mel Gomes dos Santos, Ana Luiza Silva-Araújo, Pamella Luize Peres-Damásio, Valéria Gonçalves de Alvarenga, Luciana Souza de Oliveira, Eladio Flores Sanchez, Carlos Chávez-Olórtegui, Luana Silveira da Rocha Nowicki Varela, Ana Luiza Bittencourt Paiva, Clara Guerra-Duarte

**Affiliations:** 1 Molecular Toxinology Lab, Research and Development Department, Ezequiel Dias Foundation—FUNED, Belo Horizonte 30510-010, MG, Brazil; rafaelasmagalhaes@hotmail.com (R.S.-M.); aylamelsantos@hotmail.com (A.M.G.d.S.); analu03sa@gmail.com (A.L.S.-A.); pamelladamasio1@gmail.com (P.L.P.-D.); analuiza.paiva@funed.mg.gov.br (A.L.B.P.); 2 Animal Venoms Biochemistry Lab, Research and Development Department, Ezequiel Dias Foundation—FUNED, Belo Horizonte 30510-010, MG, Brazil; valeria.alvarenga@funed.mg.gov.br (V.G.d.A.); luciana.oliveira@funed.mg.gov.br (L.S.d.O.); eladiooswaldo@gmail.com (E.F.S.); 3 Protein Imunochemistry Lab, Institute of Biological Sciences, Federal University of Minas Gerais—UFMG, Belo Horizonte 31270-901, MG, Brazil; olortegi@icb.ufmg.br; 4 Arachnid Proteomics Lab, Research and Development Department, Ezequiel Dias Foundation—FUNED, Belo Horizonte 30510-010, MG, Brazil; luana.varela@funed.mg.gov.br

**Keywords:** *Loxosceles*, spider venom, hemolysis, platelet aggregation, fibrinogenolysis

## Abstract

Spiders of the genus *Loxosceles* represent a public health problem in Brazil due to the severity of the cutaneous and systemic effects that may result from their bite. In the systemic form of loxoscelism, hemolytic anemia, thrombocytopenia, and disseminated intravascular coagulation can occur. Despite the seriousness of *Loxosceles* accidents, the venom of some species has not yet been properly characterized considering these hemotoxic effects, such as that of *Loxosceles amazonica*, *Loxosceles aff. Variegata*, and *Loxosceles similis*. To better understand their toxic potential, this study aimed to characterize the hematotoxic properties of these *Loxosceles* venoms. The crude venom was obtained from specimens of *L. amazonica*, *L. aff. Variegata*, and *L. similis* available from Funed’s arachnidary. In washed platelets, *L. aff. variegata* inhibited platelet aggregation induced by collagen and convulxin, whereas *L. amazonica* and *L. similis* venoms were able to induce platelet aggregation. In the in vitro hemolysis assays, all venoms experimentally induced direct hemolysis of human erythrocytes in a concentration-dependent manner, with different intensities. Furthermore, evidence suggest that the ABO and Rh systems may influence hemolytic activity. Finally, the studied *Loxosceles* venoms degraded fibrinogen, suggesting possible alterations in the coagulation cascade. Based in the here-presented preliminary study, in vivo assays in model animals are needed to verify the real toxic potential of these species’ venom, building up knowledge to elucidate the action of *Loxosceles* venoms in blood.

## 1. Introduction

The spider’s genus *Loxosceles*, which are part of the *Sicariidae* family, currently comprise 170 described species, widely distributed throughout the world, especially in South America [1]. In the areas where these spiders are found, they are considered a public health problem, since *Loxosceles* accidents can cause skin manifestations, such as pain and necrosis, to severe systemic symptoms, such as fever, malaise, hemolytic anemia, thrombocytopenia, and disseminated intravascular coagulation, with acute kidney injury (AKI) being the main cause of death in cases of loxoscelism [2,3,4].

In Brazil, accidents involving *Loxosceles* spiders are more common in the South region, typically caused by either *Loxosceles intermedia*, *Loxosceles laeta*, or *Loxosceles gaucho* [5]. However, loxoscelism is on the rise in other regions of Brazil [6,7]. The venom of Brazilian *Loxosceles* species with occurrence other than in the South region have not yet been fully studied, as they were not considered of medical relevance. Nevertheless, the increasing incidence and reported severe cases within these regions [8,9] justify further investigation of these venoms’ toxic potential.

*L. amazonica* is a species that has been recorded both in the peridomestic and intradomiciliary environment in the northern, northeastern, and midwestern regions of Brazil, in the states of Amazonas, Pará, Tocantins, Bahia, Ceará, Maranhão, Paraíba, Pernambuco, Piauí, and Mato Grosso, with an isolated case of the accident reported in Ceará [10,11,12]. The venom of *L. amazonica* exhibit complement-dependent hemolytic activity, with phospholipases D identified as the primary contributors. Notably, *L. amazonica* venom does not induce keratinocyte cell death and possesses sphingomyelinase activity similar to that of *Loxosceles laeta*, a species of medical relevance in Brazil [7,13]. Furthermore, *L. amazonica* venom is effectively recognized by therapeutic antivenoms and anti-phospholipase D antibodies. Its venom demonstrates sphingomyelinase D, gelatinase, and hyaluronidase activities in vitro, all of which are inhibited by therapeutic antivenom [7].

*Loxosceles variegata* is a species officially registered in Paraguay and Argentina. *Loxosceles* spiders similar to this species have been reported in Brazil in the state of Minas Gerais [1,14,15,16]. Many of these individuals have been collected in the municipality of Ituiutaba in the state of Minas Gerais, including those used in the present study. However, despite having similar characteristics to *L. variegata*, significant morphological differences were also found. Thus, a better taxonomic description of the species found in Ituiutaba is needed to confirm it as *L. variegata* or as a new species within the genus *Loxosceles*. Considering this, we chose to refer to the specimens studied here as *Loxosceles aff. variegata*, in which the abbreviation aff., from affinis, defines taxonomic terminology used to indicate that a species mentioned is related but not identical to the species in the binomial that is demonstrated.

*Loxosceles similis*, described in 1898 as a species native to São Paulo, was later identified in the Serra da Bodoquena, Mato Grosso do Sul, where it predominates in natural environments and was first considered as a non-synanthropic species [17,18], being often found in caves also in Minas Gerais, Pará, and Bahia [17,19]. Despite this first assessment, *L. similis* has been widely found in the city of Belo Horizonte, in synantropy [20]. Specimens of this species have been also found in domiciles in other cities of this state, such as Nova Lima, Caeté, Imbé de Minas, and São João Del Rey, according to the analysis of individuals brought by the population to the Arachnidary of Fundação Ezequiel Dias. The venom of *Loxosceles similis* has experimental biological activities similar to those of the spiders *Loxosceles laeta*, *L. gaucho*, and *L. intermedia* [21].

*Loxosceles* venom contains a complex mixture of toxic proteins, most notably phospholipases D (PLD). This toxin contributes to a cascade of inflammation, dermonecrosis, platelet aggregation, hemolysis, and nephrotoxicity [22]. Studies indicate that phospholipase D induces platelet activation and aggregation by producing lysophosphatidic acid, which stimulates receptors on the cell membranes of human platelets [23]. In addition, Loxosceles venom toxins have been shown to have hemolytic activity, directly affecting human erythrocytes and causing hemolysis through the metabolism of phospholipids such as sphingomyelin and lysophosphatidylcholine [23].

**Figure 1:**
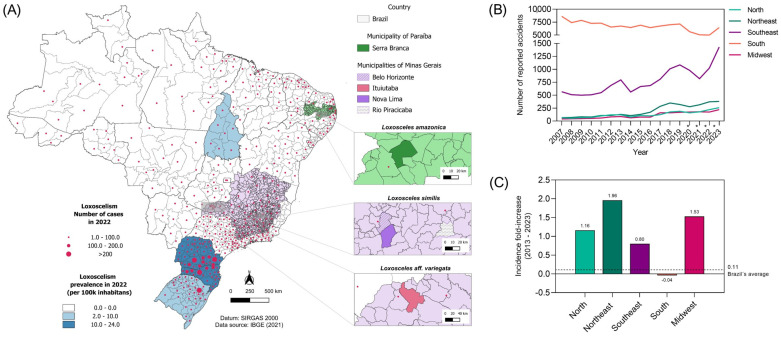
Reported accidents involving *Loxosceles* spiders in Brazil (2007–2022) and locations of spider specimen collections. (**A**) Map of Brazil depicting the occurrence of *Loxosceles*-related accidents by state in 2022, along with the regions where the species used in this study were collected. (**B**) The number of reported accidents caused by *Loxosceles* spiders from 2007 to 2023, categorized by region within Brazil. (**C**) The change in the incidence rate of *Loxosceles* accidents over a decade (2013–2023) per region, with the national average represented by a dotted line. Color intensity reflects incidence rates as detailed in the legend. The map was created using QGIS 3.32.3 software. Incident data was retrieved from the Sistema de Informação de Agravos de Notificação—SINAN. * Data is subject to revision by SINAN for the period 2020–2023. Data from the Southeast region does not include Espirito Santo state, which stopped providing data to SINAN since 2020.

Loxoscelism is a public health problem, but its prevalence is still unknown or underestimated in most Brazilian states due to underreporting of cases and the low understanding of the interaction of these spiders with the human population [11]. Data on accidents involving spiders, and more specifically spiders of the Loxosceles genus, are compiled each year by a Notifiable Diseases Information System (SINAN), linked to the Brazilian Ministry of Health. According to the data extracted from this platform (Figure 1B), in the regions where the species studied in this work occur (North and Northeast for *L. amazonica* and Southeast for *L. aff.variegata* and *L. similis*—Figure 1A) there is an annual increase in the number of reported cases.

During the decade from 2012 to 2022, the incidence of *Loxosceles* accidents more than doubled in the Midwest and Northeast regions (Figure 1C). There were also significant increases in the North and Southeast, whereas the South saw a decrease in incidence (Figure 1C). It is important to highlight that the absolute number of reported cases and the overall prevalence of loxoscelism in all other Brazilian regions remain lower than in the South, which has consistently reported the highest number of cases (Figure 1B). Although the absolute number of reported accidents is notably lower than those reported in the southern region (Figure 1A—Centroids), it is necessary to better understand the potential for damage of the species typical of the other regions that have shown an increase in the number of accidents.

Considering the scarcity of information concerning the toxic potential of *Loxosceles* spiders from other regions of Brazil, the aim of this study is to characterize the hemotoxic properties of the venoms of *Loxosceles amazonica*, *Loxosceles aff. Variegata*, and *Loxosceles similis*, evaluating the direct hemolytic activity on human erythrocytes. In addition, the study will investigate the action of the venoms on platelets and the platelet-aggregation process, as well as the ability of the venoms to degrade fibrinogen. With this, a deeper understanding of the toxic potential of *Loxosceles* venoms will be sought.

## 2. Results and Discussion

### 2.1. Platelet Aggregation and Inhibition Assays

Loxoscelism represents a public health concern in Brazil due to its increasing incidence in some regions. Among its pathophysiological effects, the most severe systemic manifestations are intravascular hemolysis, disseminated intravascular coagulation, thrombocytopenia, and acute kidney injury [3,24,25].

**Figure 2:**
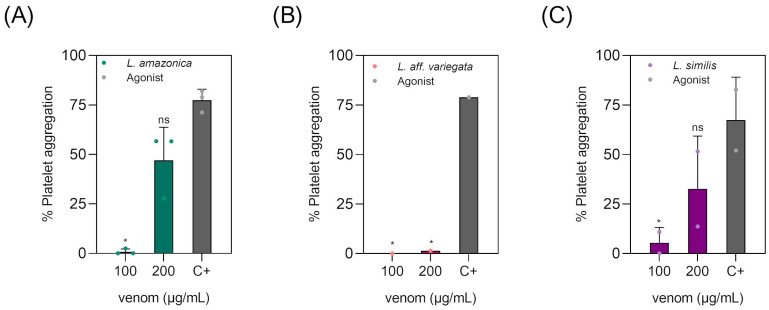
The crude venom of *Loxosceles amazonica* and *Loxosceles similis* induce platelet aggregation. Washed platelets were incubated with 100 μg/mL and 200 μg/mL of *Loxosceles* crude venoms. Aggregation was monitored by measuring light transmittance for 10 min by an aggregometer. The percentage of aggregation was automatically calculated by comparing the initial optical density with the optical density after the addition of the aggregating agent, using the HemoRam 1.1 software. The mean ± standard deviation is shown. The results are representative of two or three experiments with different individual donors (points of graph). (**A**) Platelet aggregation with *Loxosceles amazonica*; (**B**) *Loxosceles aff. Variegata*, and (**C**) *Loxosceles similis*. Collagen or convulxin were used as platelet-aggregation agonists (C+). Statistical analysis was performed using one-way ANOVA (Kruskal–Wallis test) with Dunn post-test for multiple comparison. (*) = *p* ≤ 0.05.

Thus, to verify the action of the venom of understudied *Loxosceles* species on human platelets, we performed platelet aggregation and inhibition assays using washed platelets. *L. amazonica* venom induced spontaneous platelet aggregation, when 200 µg/mL of crude venom was used (Figure 2A), as well as *L. similis* (Figure 2C). This physiological alteration resulting from envenoming has already been observed in other *Loxosceles* species of medical concern, such as *L. intermedia*, *L. gaucho*, and *L. reclusa* [2,26,27]. This activity was not observed for the venom of *L. aff. variegata* (Figure 2B). A pilot assay, using platelet-rich plasma (PRP) instead of washed platelets and the same venoms in 100 µg/mL concentration, mirrored this same result (Supplementary Figure S1a). This indicated that the venoms from *L. amazonica* and *L. similis* act directly in platelets, dismissing other plasma components to induce aggregation.

Studies have identified phospholipase D (PLD) enzymes as the primary molecules responsible for platelet aggregation in *Loxosceles* venoms [2,26,28,29]. These toxins play a central role in venom toxicity, being chiefly responsible for its deleterious effects, including dermonecrosis, hemolysis, and platelet aggregation. More recently, beyond the indirect effects mediated by factors released through PLD activity, it has been demonstrated that a recombinant PLD from *Loxosceles gaucho* venom, LgRec1, can directly bind to platelets without requiring other plasma components, although these components are ultimately essential for full platelet aggregation. Additionally, it was shown that LgRec1 induces the exposure of phosphatidylserine (PS) on the platelet membrane, potentially enhancing platelet aggregation [23]. Despite these findings, the specific platelet receptor targeted by PLDs remains unidentified, and the mechanism underlying platelet aggregation induced by *Loxosceles* venom is yet to be fully elucidated.

**Figure 3:**
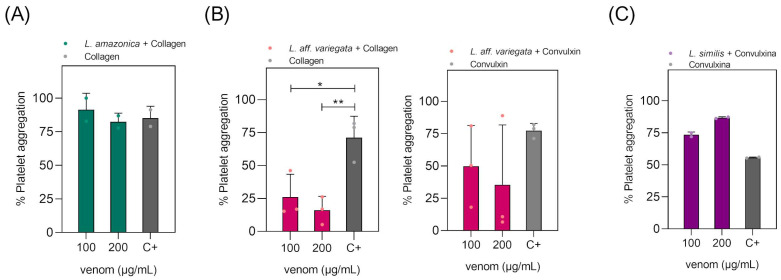
The crude venom of *Loxosceles aff. variegata* inhibits platelet aggregation induced by collagen and convulxin. Washed human platelets were pre-incubated with different concentrations of *Loxosceles aff. variegata* venom (100 and 200 μg/mL) under agitation at 600 rpm at 37 °C. After 3 min, platelet aggregation was induced by 10 μg/mL collagen or 0.3 mg/mL convulxin and monitored by aggregometer by measuring light transmittance for 7 min. The mean ± standard deviation is shown. The results are representative of three experiments with different individual donors (points of graph). (**A**) The crude *L. amazonica* venom does not have the ability to inhibit platelet aggregation induced by agonist collagen. (**B**) Platelet aggregation assay to assess the ability of crude *Loxosceles aff. variegata* venom to inhibit collagen-induced and convulxin-induced aggregation. (**C**) The crude *L. similis* venom does not have the ability to inhibit platelet aggregation induced by agonist Convulxin. Statistical analysis was performed using two-way ANOVA with Tukey post-test for multiple comparison. (*) = *p* ≤ 0.05 and (**) = *p* ≤ 0.01.

Conversely, the venom of *L. aff. variegata* had a potential to inhibit washed platelet aggregation previously induced by an agonist (either collagen or convulxin) when the crude venom was used at concentrations of 100 µg/mL and 200 µg/mL (Figure 3B) but had a greater inhibitory potential to reduce Collagen-induced platelet aggregation when compared to the inhibition using the agonist Convulxin. This property was not observed for *L. amazonica* and *L. similis* venom (Figure 3A,C). In the aggregation inhibition assay using PRP, 100 µg/mL of each venom, and ADP as the aggregation inducer, all tested venoms, including *L. aff. variegata*, were unable to reverse platelet aggregation, contrary to the findings observed with washed platelets (Supplementary Figure S1a). This suggests that *L. aff. variegata* venom may contain components that counteract GPIV or alfa2beta1 integrin agonists (collagen and convulxin), but not purinergic ones (ADP).

### 2.2. Dose-Response Curve of the Direct Hemolytic Activity of Loxosceles Venoms

**Figure 4:**
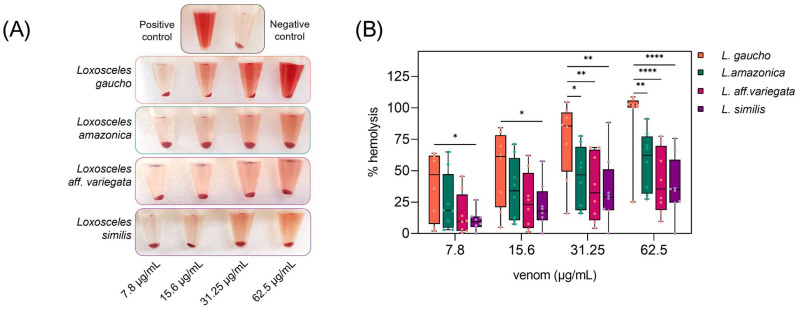
In vitro hemolytic assay of *Loxosceles* venoms. Human erythrocytes were exposed to various concentrations (7.8; 15.6; 31.25; and 62.5 μg/mL) of different *Loxosceles* spider venoms for 20 h at 37.0 °C and were evaluated for hemolysis. As a negative control, the erythrocytes were incubated only in Ringer’s Lactate. The positive control was incubated with a 0.1% (*v*/*v*) Triton X-100 solution. (**A**) The tubes containing the Ringer’s Lactate solution, the venoms, and the controls after incubation and centrifugation. (**B**) Percentage of hemolysis considering Triton X-100 (positive control) as 100%. Statistical analysis was performed using two-way ANOVA with Tukey post-test for multiple comparison. (*) = *p* ≤ 0.05, (**) = *p* ≤ 0.01 and (****) = *p* ≤ 0.0001.

To evaluate the hemolytic capacity of *Loxosceles* venoms on human erythrocytes, experiments were performed to assess their ability to trigger direct hemolysis of human erythrocytes in vitro. Erythrocytes incubated only with Ringer’s Lactate, where no hemolysis should be observed, were used as a negative control. The experiment was conducted with at least eight different samples, including all possible blood types, regarding the ABO/Rh systems. In this assay, we used *Loxosceles gaucho* venom as a control. This spider is considered medically significant in Brazil, and the hemolytic activity of its venom has already been well-documented [27]. The results of the experiments (Figure 4A) show a visible difference in hemolytic activity between species, with *L. gaucho* and *L. similis* triggering the most and least hemolysis, respectively.

It is known that PLDs can induce hemolysis both in the presence (indirect hemolysis) and absence of serum (direct hemolysis) [30,31]. Magalhães et al. (2013) [27] showed that a recombinant phospholipase D (PLD) from *L. gaucho*, called LgRec1, as well as the spider’s crude venom, caused only mild hemolysis (around 20%) at a concentration of 25 µg/mL. In the study by Fukuda et al. (2017) [23], LgRec1 bound and unbound to EGFP (Enhanced Green Fluorescent Protein) was tested, both in the presence and absence of serum. The results showed that both forms of the toxin could promote hemolysis, although direct hemolysis was more prominent only after 12 h of exposure. However, notable differences in hemolysis values were observed when comparing the effects of *L. gaucho* venom at different concentrations. In a complementary way, the study by Chaves-Moreira (2009) [31] provided evidence that PLD from *L. intermedia* venom can trigger direct hemolysis in human blood cells, reinforcing the role of these toxins in the mechanism of hemolytic action in different *Loxosceles* species.

The results presented here show that the tested *Loxosceles* venoms possess direct hemolytic activity on human erythrocytes, as in the experimental conditions used there was no participation of the complement system in the process of cell lysis since the assay was performed in the absence of plasma. Complement- dependent hemolysis has already been seen for *L. intermedia* by Moreira (2008) [31] and for *L. amazonica* by Lopes et al. (2021) [13]. Furthermore, the hemolytic effect was shown to be directly proportional to the amount of venom used. The *Loxosceles* species studied in the present work are not typically considered of medical concern in Brazil. However, the obtained results points to a relevant toxicity upon human blood, indicating that their venoms have the potential to cause significant harm.

### 2.3. Assessment of Rh System Interference in Direct Hemolysis

We observed a great variability in the hemolysis assay between the blood samples from different donors. Therefore, the data obtained was re-analyzed, considering the different blood types, at the 31.25 µg/mL concentration. The aim of this analysis was to verify the hypothesis that the antigens of the ABO and Rh systems could interfere with the hemolytic activity of *Loxosceles* venoms. We analyzed one sample of A-blood, two of A+, one of B-, one of B+, one of AB-, one of AB+, two of O+, and two of O-, all confirmed by blood typing. Due to the rarity of some blood types and the limited amount of the spider venoms, we were unable to perform a more conclusive assessment, but we believe that the obtained preliminary data can help to elucidate the spider venom hemolysis dependency on blood antigens. This knowledge can be helpful in analyzing the risks of aggravation in human loxoscelic accidents.

**Figure 5:**
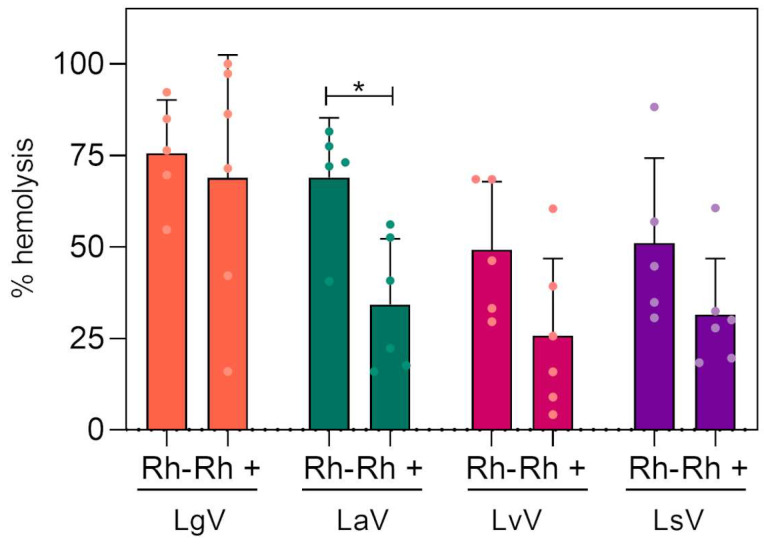
Influence of the Rh system on the direct hemolytic activity of *Loxosceles* venoms. The blood types were incubated with 31.25 μg/mL of different *Loxosceles* venoms for 20 h. LaV—*Loxosceles amazonica* venom. LgV—*Loxosceles gaucho* venom. LvV—*Loxosceles aff. variegata* venom. LsV—*Loxosceles similis* venom. Statistical analysis was performed using two-way ANOVA, with post-test of Bonferroni. (*) = *p* < 0.05.

Figure 5 shows that Rh-negative erythrocytes are more susceptible to hemolysis induced by *Loxosceles* venoms than Rh-positive ones. Due to the limited number of samples, the results were statistically significant (*p* < 0.05) only for the *L. amazonica* venom, although there was a tendency in all groups. This effect may be related to the action of phospholipases D, present in the venoms, which degrade the phospholipids present in cell membranes, including erythrocytes, resulting in hemolysis [30,32]. Thus, the absence of the Rh antigen on Rh-negative erythrocytes may facilitate the interaction of the venom with the cell membrane, making them more vulnerable to the action of phospholipases D.

In contrast, [33] study on *Loxosceles intermedia* found no significant differences in hemolysis between Rh-positive and Rh-negative erythrocytes, suggesting that different *Loxosceles* species may have different hemolytic activities. These results reinforce the hypothesis that the Rh system plays a role in modulating the hemolysis caused by *Loxosceles* venoms, with Rh-negative erythrocytes being more vulnerable. However, more studies and a larger number of samples are needed to explore these interactions in more detail.

When grouping the data according to the ABO system, it was found that blood types with more antigens on the erythrocyte membrane, such as AB+, showed lower levels of hemolysis when compared to types A+, O+, and B+. An exception was type B+ blood, which exhibited greater hemolysis when incubated with *L. gaucho* venom. Among Rh-negative erythrocytes, the highest rates of hemolysis were observed in type O-, followed by AB-, A-, and B-. However, these results need to be confirmed with a larger number of samples, as in some cases there was only one donor for certain blood types (as shown in the Supplementary data Figure S2).

### 2.4. Fibrinogenolytic Activity

**Figure 6:**
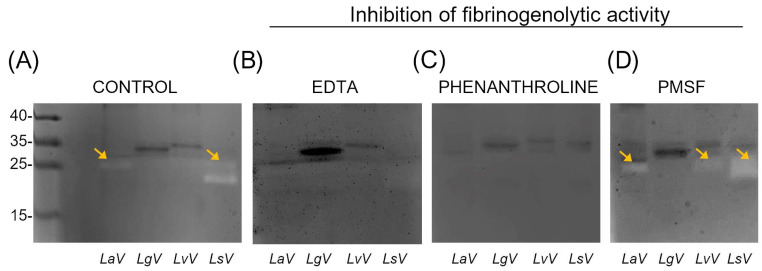
Zymogram of the fibrinogenolytic activity of *Loxosceles* venoms and the inhibition of this activity. (**A**) Zymography of *Loxosceles* venoms using 12% SDS–PAGE, containing 5 mg/mL of bovine fibrinogen integrated into the gel. (**B**) Gel incubated overnight in a 1 M phosphate buffer at pH 8.0 with 2 mM EDTA. (**C**) Gel incubated overnight in a 1 M phosphate buffer at pH 8.0 with 3 mM Phenanthroline. (**D**) Gel incubated overnight in a 1 M phosphate buffer at pH 8.0 with 5 mM Phenylmethylsulfonyl Fluoride (PMSF). Arrows in yellow point to regions where enzymatic degradation by the venoms is present. LaV—*Loxosceles amazonica* venom. LgV—*Loxosceles gaucho* venom. LvV—*Loxosceles aff. variegata* venom. LsV—*Loxosceles similis* venom.

To verify whether the *Loxosceles* venoms of the studied species have proteolytic activity on fibrinogen, a fibrinogen-degradation assay was performed in two different formats. In the zymogram, fibrinogen was embedded in the polyacrylamide gel and after the run, it was allowed to sit overnight in a phosphate buffer solution with a pH of 8.0 for the reaction to occur. Figure 6A illustrates clear areas indicating enzymatic degradation caused by the venoms, thereby confirming the presence of proteolytic activity and proteinases in the venoms, especially in the venoms of *L. amazonica* and *L. similis*. The observed clear bands are around ~25 kDa, a molecular weight compatible with *Loxosceles* Astacin-like proteinases (LALP) [34]. Treatment of the gel with EDTA and Phenanthroline (Figure 6B,C), both metalloprotease inhibitors, led to a reduction in proteinase activity. In contrast, when treating the gel with PMSF (serine-protease inhibitor), there was no inhibition of proteolytic activity (Figure 6D).

In the direct assay, for the control sample, it is possible to see in the SDS–PAGE the three polypeptide chains of fibrinogen, the alpha (α), beta (β), and gamma (γ) chain as separate bands after gel staining. The change in density of these bands after incubation of venom with fibrinogen may indicate the presence of proteolytic enzymes in the venoms. Most enzymes with fibrinogenolytic action preferentially degrade the α subunit, followed by β, as has been seen for snake venoms [35] for *Loxosceles laeta* [36,37,38] and for *Loxosceles reclusa* [38].

**Figure 7:**
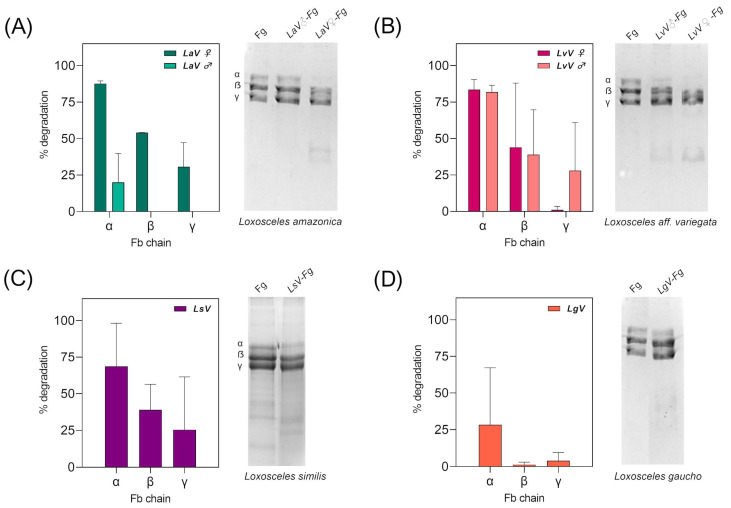
Fibrinogenolytic activity of Brazilian *Loxosceles*. Proteolytic activity was determined by a fibrinogen digestion (Fg) assay described by Medina-Santos et al., 2019. Fibrinogenolytic activity was performed using 3 µg of *Loxosceles* venoms, incubated with bovine or human fibrinogen for 16 h at 37 °C. The fibrinogen samples, either pure or pre- incubated with the venoms, were analyzed by 12% SDS–PAGE. The graphs were plotted with the mean and standard deviation of the percentage density of the bands compared to the fibrinogen control, considered as 100%, analyzed in ImageJ, considering two independent assays. (**A**) On the left, the graph shows the percentage of degradation of fibrinogen chains. On the right, 12% SDS–PAGE displays the fibrinogenolytic activity of male and female *L. amazonica* venom. (**B**) On the left, the graph shows the percentage of degradation of fibrinogen chains. On the right, 12% SDS–PAGE displays the fibrinogenolytic activity of male and female *L. aff. variegata* venom. (**C**) On the left, the graph shows the percentage degradation of fibrinogen chains. On the right, 12% SDS–PAGE displays the fibrinogenolytic activity of *L. similis* venom. (**D**) On the left, the graph shows the percentage degradation of fibrinogen chains. On the right, 12% SDS–PAGE displays the fibrinogenolytic activity of *L. gaucho* venom. LaV—*Loxosceles amazonica* venom. LgV—*Loxosceles gaucho* venom. LvV—*Loxosceles aff. variegata* venom. LsV—*Loxosceles similis* venom. Fg—Fibrinogen.

Figure 7 shows the results of the fibrinogenolytic activity assay of the venoms. It can be observed the degradation of the α and β subunit of fibrinogen by the venoms from *L. amazonica* (Figure 7A) and *L. aff. variegata* (Figure 7B) after 16 h of incubation. The pooled venom from *L. similis* (Figure 7C) and *L. gaucho* (Figure 7D) also demonstrated fibrinogenolytic activity, although less prominently than the other two venoms.

## 3. Conclusions

The venoms from *L. amazonica* and *L. similis* were able to induce platelet aggregation, and *L. aff. variegata* venom was able to inhibit platelet aggregation induced by the agonists tested. In the in vitro hemolysis experiments, the venoms of *Loxosceles* species were capable of triggering direct hemolysis of human erythrocytes. The ABO and Rh systems seem to influence the degree of hemolytic activity of the venoms on human erythrocytes. However, due to the small sample size of each ABO blood type, further analyses will be needed to confirm its influence on the hemolytic activity of the venom. Finally, all the studied loxoscelic venoms degraded fibrinogen, suggesting possible alterations in the coagulation cascade, which may lead to intravascular hemolysis or disseminated intravascular coagulation, the main signs of systemic loxoscelism. This study has increased knowledge regarding the activities of the venoms of *L. amazonica*, *L. similis*, and *L. aff. variegata* species. However, in vivo studies are needed to verify the true toxic potential and elucidate the role of these loxoscelic venoms in hemostasis.

## 4. Materials and Methods

### 4.1. Spiders, Venoms, and Antivenoms

Specimens of *L. amazonica* were collected in Serra Branca municipality, Paraíba state; *L. aff. variegata* was collected in Ituiutaba, Minas Gerais state; and *L. similis* was collected in Belo Horizonte, Nova Lima, and Rio Piracicaba, all in Minas Gerais state (Figure 1) (License SISBIO: 21102-9 and 21102-11, SISGEN: A651423). All spiders were identified and kept in the Arachnidarium of Fundação Ezequiel Dias (FUNED). Venom was obtained by microdissection of the venom glands, with spiders previously anesthetized at −20 °C for 3 min. After extracting the glands, they were macerated and then centrifuged at 12,000 rpm for 10 min. The supernatant was then collected and kept at −80 °C until needed.

Venom from *Loxosceles gaucho*, used as a control for some experiments, was obtained by electrostimulations and provided by Centro de Produção e Pesquisa em Imunobiológicos (CPPI), Paraná, Brazil. The protein concentration was determined using the DC Protein Assay from Bio-Rad, using bovine serum albumin as a standard.

### 4.2. Platelet Aggregation and Inhibition Assay

After approval by the Research Ethics Committee (CEP/FUNED—CAAE: 52987121.4.0000.9507), human blood from NSAIDs-free healthy donors was collected in vacuum tubes containing acid-citrate-dextrose (ACD), and to obtain washed platelets, the initial centrifugation is 1300 rpm, 15 min at 37 °C. To evaluate the effects of the crude venoms studied on platelet aggregation, human platelets were isolated and washed according to Sanchez et al. (2016) [39]. At the end of washing, platelets were resuspended in Tyrode pH = 7.4, and the concentration of platelets was adjusted to 2.5 × 10^5^ platelet/μL.

For the platelet-aggregation assay, 225 µL of the washed platelets were incubated with 100 and 200 μg/mL of the crude venoms, and aggregation was monitored by measuring transmittance on an AggRAM platelet aggregometer (Remote Aggregation Analyzer, Helena Laboratories, Beaumont, TX, USA) under 600 rpm agitation at 37 °C for 10 min.

For platelet-inhibition assays, 225 μL of washed platelets were pre-incubated with 100 and 200 μg/mL of the venoms in Tyrode’s pH 7.4 solution for 3 min. Subsequently, platelets were stimulated by the addition of agonists: either 10 µg/mL of Collagen or 0.3 mg/mL of Convulxin. Aggregation monitoring was performed under the same conditions as described previously in the AggRAM platelet aggregometer. The assays were performed in three independent experiments, with negative controls (only washed platelets) and positive controls (washed platelet with agonists). Both results were analyzed using the equipment’s software HemoRam, version 1.1.

### 4.3. Hemolytic Activity

Human blood from healthy donors was collected in vacuum tubes containing ethylenediaminetetraacetic acid (EDTA), and blood type was confirmed using monoclonal anti-A (Lorne laboratories limited, Berkshire, UK), anti-B (Lorne laboratories limited), and monoclonal anti-D antibodies, and the negative control Rh (ASEM-NPBI Produtos Hospitalares Ltd., Itapecerica da Serra, São Paulo, Brazil). After determining the donor’s blood type, the sample was centrifuged at 170× *g* for 15 min to remove platelet-rich plasma. The remaining red cells were washed three times with Ringer’s Lactate solution (0.6% NaCl, 0.03% KCl, 0.02% CaCl_2_, 0.31% sodium lactate) and centrifuged at 2000 rpm for 3 min between washes. Later, the washed erythrocytes were resuspended in Ringer’s Lactate at a final concentration of 5 × 10^8^ cells/mL. A volume of 200 μL of washed red blood cells (1 × 10^8^) were added to each Eppendorf tube containing 200 μL of each serial dilution of the venoms (62.5 to 7.8 µg/mL). Assays were performed in duplicate for each blood sample, having a total of eight different samples per venom, with negative (red blood cells in Ringer’s Lactate) and positive controls (red blood cells in 0.01% (*v*/*v*) Triton X-100). After 20 h of incubation with gentle shaking, controls and samples were centrifuged for 5 min at 1000 rpm, and the absorbance of the supernatants was read at 550 nm (Multiskan SkyHigh Microplate Spectrophotometer, Thermo Scientific Waltham, MA, USA). The absorbance values were converted to percent hemolysis using the absorbance values of the positive control as 100% of erythrocyte lysis [31,40].

### 4.4. Fibrinogenolytic Activity

#### 4.4.1. Zymogram

The zymogram was executed using a 12% SDS–PAGE gel with 5 mg/mL of bovine fibrinogen added before polymerization. The venoms were diluted with a sample buffer under non-reducing conditions and then run through electrophoresis, starting at 50 V for the first hour and then increasing to 100 V until completion, all in a chilled environment. After the run, the gels were washed in 2.5% (*v*/*v*) Triton X-100 for 1 h to remove SDS from the gel, then incubated overnight at 37 °C in 1 M phosphate buffer pH 8.0 and stained with Coomassie blue. The clear regions of the substrate, contrasting with the blue-stained background, signify enzyme degradation, indicating the presence of fibrinogenolytic activity. For the inhibition assays, after washing with 2.5% (*v*/*v*) Triton X-100, the gel was incubated separately either with 2.0 mM EDTA, 5.0 mM Phenylmethylsulfonyl Fluoride (PMSF), or 3 mM 1,10 phenanthroline to evaluate the proteolytic family responsible for the fibrinogen degradation [41].

#### 4.4.2. Fibrinogen Digestion Assay

The fibrinogenolytic activity of the venoms was determined by a direct fibrinogen-digestion assay as described by Medina-Santos (2019) [36], with modifications. An amount equivalent to 3 µg of venom was added to 50 µL of a solution containing 2.5 mg/mL of bovine fibrinogen diluted in 25 mM Tris-HCl buffer containing 0.15 M NaCl (pH 7.4) and incubated for 16 h at 37 °C. After this period, 50 μL of denaturing solution (10 M urea, 4% β-mercaptoethanol, 4% SDS) was added, and this mixture was incubated at room temperature for another 16 h. Then, 10 μL of the sample was added to 10 μL of sample buffer under reducing conditions and analyzed by SDS–PAGE on a 12% polyacrylamide gel, subsequently stained with Coomassie blue. To estimate fibrinogen degradation by venom, the density of fibrinogen bands was measured by pixel quantification using ImageJ 1.51 software.

### 4.5. Statistical Analysis

Statistical analysis was conducted using GraphPad Prism (version 9.0). Normality of the data was assessed via the Shapiro–Wilk test, with data sets considered parametric if *p*-values exceeded 0.05. For non-parametric data, one-way ANOVA (Kruskal–Wallis test) followed by Dunn’s post-test for multiple comparisons was used. For parametric data, two-way ANOVA with Tukey’s post-test for multiple comparisons was employed. A *p*-value of less than 0.05 was considered statistically significant in all tests. Each assay was performed in duplicate and repeated in at least two independent experiments. Results are expressed as means with standard deviations.

## Data Availability

The original contributions presented in this study are included in the article/supplementary material. Further inquiries can be directed to the corresponding author(s).

## Supplementary Materials

The following supporting information can be downloaded at: https://www.mdpi.com/article/10.3390/toxins16120532/s1, Figure S1: Platelet aggregation and inhibition assays with platelet-rich plasma. Figure S2: Influence of the ABO and Rh systems on the direct hemolytic activity of Loxosceles venoms.
