# Supplementary material for: Venom from Loxosceles Spiders Collected in Southeastern and Northeastern Brazilian Regions Cause Hemotoxic Effects on Human Blood Components
Source: Toxins (Basel). 2024 Dec 10;16(12):532. doi: 10.3390/toxins16120532 (PMC11680057; doi:10.3390/toxins16120532)
Supplement: Supplementary file 1 [file toxins-16-00532-s001.zip › toxins-3312881-supplementary.pdf]

Supplementary Material: Venom from *Loxosceles* Spiders Collected in Southeastern and Northeastern Brazilian Regions Cause Hemotoxic Effects on Human Blood Components

Rafaela Silva-Magalhães, Ayla Mel Gomes dos Santos, Ana Luiza Silva-Araújo, Pamella Luize Peres-Damásio, Valéria Gonçalves de Alvarenga, Luciana Souza Oliveira, Eladio Flores Sanchez, Carlos Chavez-Olortegui, Luana Silveira da Rocha Nowicki Varela, Ana Luiza Bittencourt Paiva and Clara Guerra-Duarte

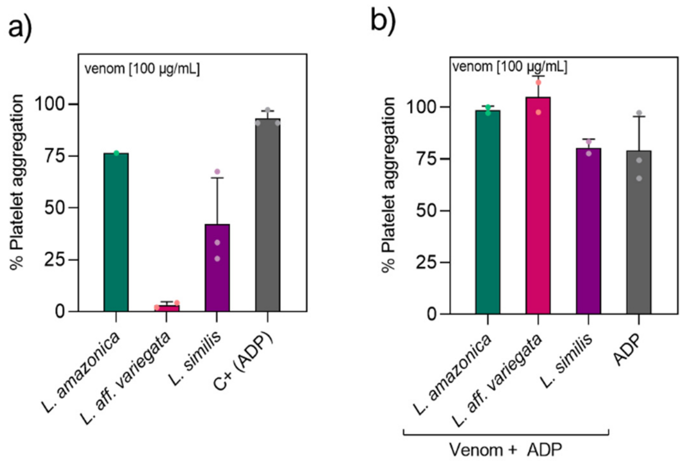

Figure S1. Platelet aggregation and inhibition assays with platelet-rich plasma. (**a**) Platelet-rich plasma was incubated with 100 µg/mL of *Loxosceles* crude venoms. Aggregation was monitored by measuring light transmittance for 10 minutes by an aggregometer. The percentage of aggregation is automatically calculated by comparing the initial optical density with the optical density after the addition of the aggregating agent, using the AggRAM software. (**b**) Platelet-rich plasma was pre-incubated with 100 µg/mL of the *Loxosceles* venoms under agitation at 600 rpm at 37 °C. After 3 min, platelet aggregation was induced by 10.0 µM ADP and monitored by aggregometer by measuring light transmittance for 7 min. The mean ± standard deviation is shown. The results are representative of two experiments with different individual donors.

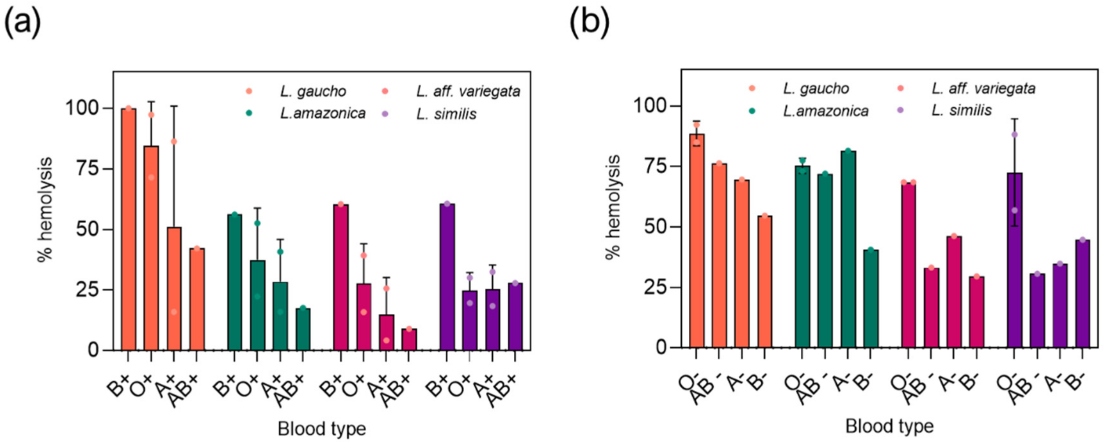

Figure S2. Influence of the ABO and Rh systems on the direct hemolytic activity of *Loxosceles* venoms. The blood types were incubated with 31.25 µg/mL of different *Loxosceles* venoms for 20 hours. As a negative control, the erythrocytes were incubated only in ringer's lactate. (**a**) Influence of the ABO and Rh-positive systems on the direct hemolytic activity. (**b**) Influence of the ABO and Rh-negative systems on the direct hemolytic activity.
